# The LIM-Only Protein FHL2 Attenuates Lung Inflammation during Bleomycin-Induced Fibrosis

**DOI:** 10.1371/journal.pone.0081356

**Published:** 2013-11-18

**Authors:** Abdulaleem Alnajar, Carolin Nordhoff, Tanja Schied, Ruth Chiquet-Ehrismann, Karin Loser, Thomas Vogl, Stephan Ludwig, Viktor Wixler

**Affiliations:** 1 Institute of Molecular Virology, Centre for Molecular Biology of Inflammation (ZMBE), Westfaelische Wilhelms-University Muenster, Muenster, Germany; 2 Friedrich Miescher Institute for Biomedical Research, Basel, Switzerland; 3 Faculty of Science, University of Basel, Basel, Switzerland; 4 Department of Dermatology, Westfaelische Wilhelms-University Muenster, Muenster, Germany; 5 Institute of Immunology, Westfaelische Wilhelms-University Muenster, Muenster, Germany; Helmholtz Zentrum München/Ludwig-Maximilians-University Munich, Germany

## Abstract

Fibrogenesis is usually initiated when regenerative processes have failed and/or chronic inflammation occurs. It is characterised by the activation of tissue fibroblasts and dysregulated synthesis of extracellular matrix proteins. FHL2 (four-and-a-half LIM domain protein 2) is a scaffolding protein that interacts with numerous cellular proteins, regulating signalling cascades and gene transcription. It is involved in tissue remodelling and tumour progression. Recent data suggest that FHL2 might support fibrogenesis by maintaining the transcriptional expression of alpha smooth muscle actin and the excessive synthesis and assembly of matrix proteins in activated fibroblasts. Here, we present evidence that FHL2 does not promote bleomycin-induced lung fibrosis, but rather suppresses this process by attenuating lung inflammation. Loss of FHL2 results in increased expression of the pro-inflammatory matrix protein tenascin C and downregulation of the macrophage activating C-type lectin receptor DC-SIGN. Consequently, FHL2 knockout mice developed a severe and long-lasting lung pathology following bleomycin administration due to enhanced expression of tenascin C and impaired activation of inflammation-resolving macrophages.

## Introduction

Fibrosis is a consequence of the excessive expression and deposition of extracellular matrix (ECM) components, which can result in sclerosis and scarring of tissues. Fibrotic changes usually take place when regenerative processes have failed or long-term inflammation occurs [1–4]. During acute inflammation and tissue damage, the affected cells release large amounts of bioactive lipids and inflammatory factors that induce the proliferation of surrounding cells, and also attract and activate a large number of immune cells and fibroblasts in order to repair the injured tissue. Activated myofibroblasts are the major source of newly synthesised fibrous connective tissue, with transforming growth factor beta (TGFβ) being the major profibrotic cytokine and activator of myofibroblasts [5,6].

Idiopathic pulmonary fibrosis is a progressive and fatal fibrosing disease of the lung with unknown aetiology, but several environmental factors such as smoking, chronic microaspiration or viral infection are known to promote its development [7,8]. The disease is characterized by damage of interstitial tissue and myofibroblast transdifferentiation with exaggerated accumulation of ECM proteins leading to scarring of alveolar compartments of the lung where gas exchange occurs [4,8,9]. Available data increasingly indicate that chronic inflammation and aberrant wound healing play an important role in the development of the disease [8–11]. 

Bleomycin (BLM) is a chemotherapeutic drug with a serious side effect - the development of lung fibrosis [12]. Its intratracheal application in mice induces a transient but severe expansion of lung fibrosis that resembles fibrotic changes in humans to a certain degree, and is currently one of the best established animal models of lung fibrosis [12–14]. The mechanism of BLM-induced lung disease is complex and still insufficiently understood. As a cytostatic drug, it induces DNA strand brakes, resulting in the inhibition of cell proliferation and cell lysis [12]. However, BLM can also cause alveolar cell damage independently of its effect on DNA by inducing lipid peroxidation and increasing the apoptosis of epithelial and endothelial cells, leading to alveolar oedema and lung inflammation [13,14]. The acute lung tissue injury and ongoing inflammation are believed to be the major reason for activation and recruitment of myofibroblasts and fibrotic alterations.

FHL2 is a scaffolding protein that can interact with numerous membranes, as well as cytosolic and nuclear proteins [15]. By forming a protein complex with integrins and focal adhesion kinase, FHL2 supports the clustering of integrins and integrin-driven assembly of matrix proteins [16]. By interaction with cytosolic proteins FHL2 is involved in regulation of NF-κB and MAPK signalling cascades [17–20]. In addition to modulating signalling molecules, FHL2 shuttles between the cytosol and nucleus, acting as a cofactor in the transcription of αSMA (alpha smooth muscle actin) and some matrix proteins, but inhibiting the expression of matrix metalloproteinases [21–23] During skin wound healing, FHL2 is upregulated in activated fibroblasts, but only transiently [24]. Pro-fibrotic cytokines such as TGFβ and S1-P (*sphingosine 1-phosphate*) induce its expression, while the pro-inflammatory cytokine IL-1 downregulates FHL2 [19,25]. 

The purpose of this work was to study the potential supportive effect of FHL2 on the development of BLM-induced lung fibrosis. Surprisingly, after administering BLM, FHL2-deficient mice developed more severe lung pathology than wild type (WT) mice.

## Materials and Methods

### Bleomycin (BLM) treatment

All animal experiments were approved by the local ethics committee and performed in strict accordance with the German regulations of the Society for Laboratory Animal Science (*GV-SOLAS*) and the European Health Law of the Federation of Laboratory Animal Science Associations (FELASA). The protocols were approved by the Landesamt für Natur, Umwelt und Verbraucherschutz Nordrhein-Westfalen (LANUV-NRW), Germany. WT and FHL2-KO mice (genetic background C57Bl/6 [26]) were kept in pathogen-free conditions and two- to four-month-old mice were used. Before BLM (B2434, Sigma-Aldrich) application, animals were anaesthetised by an i.p. injection of 200 μl of a ketamine-rompun solution (equal amounts of a 2% rompun solution and a 10% ketamine solution were mixed at the ratio of 1:10) and weighed. BLM dissolved in PBS was applied intranasally. Usually a dosis of 2 U/kg of mouse body weight was applied, if not indicated otherwise. Following BLM administration the body weight was recorded twice a week during the course of the experiment. For lung analysis, animals were euthanized on different days after BLM application and the right lungs were usually fixed with 4 % paraformaldehyde for histological studies and the left lungs were minced and homogenised either in RIPA buffer for protein analysis or in RNAlate solution for qRT-PCR studies. For *in vivo* depletion of macrophages, mice were intravenously injected with clodronate-liposomes (purchased from ClodronateLiposomes.com, The Netherlands) or PBS-liposomes as control (100 µl/mouse). The next day, they were repeatedly injected with 75 µl/mouse of clodronate-liposomes or PBS-liposomes solution, but intranasally. On the following day, the mice received BLM intranasally and 5 days later, they received another dose of 75 µl/mouse of clodronate- or PBS-liposomes intranasally. 

### Isolation, stimulation and transfection of peritoneal murine macrophages

To increase the number of isolated peritoneal macrophages, mice received 0.8 ml of 4% frozen and thawed starch gel in PBS intraperitoneally (i.p.) three days before stimulation. The cells were washed out from the abdominal cavities of euthanized mice with 5 ml of sterile PBS. Cells from three to four animals were pooled and washed twice with PBS by centrifugation at 1200 rpm for 5 min at 4°C, after which they were resuspended in DMEM with 2% foetal calf serum (FCS) and 50 µg/ml gentamicin, and plated into 6-well dishes with 2x10^6^ cells per dish. After incubation at 37°C in a humidified incubator under 5% CO_2_ for 2-3 h, the non-adherent cells were washed off with PBS and the remaining attached macrophages were stimulated with lipopolysacharide (LPS, 1µg/ml) or BLM (15 U/ml) and mouse lung tissue lysate (containing carbohydrates) for an additional 24 h. 

To obtain lung lysates, lungs from two WT mice were minced, placed in 5 ml of PBS, repeatedly frozen in liquid nitrogen four times and thawed in a 37°C-water bath, before being sonicated on ice three times for 30 seconds each and centrifuged. The supernatant was then filtered sterile and used for stimulating macrophages at a dilution of 1:100. The optimal dilution was determined in preliminary experiments. The lung lysate was used as a source of released endogenous damage-associated molecular pattern molecules.

For FHL2 rescue experiments, suspensions of freshly isolated peritoneal macrophages were transfected with the pCS2+MT plasmid containing the human myc-tagged FHL2 or with empty vector using the Amaxa apparatus and the Amaxa Mouse Macrophage Nucleofector Kit (Lonza, Cologne, Germany), following the manufacturer's instructions. For each transfection set, 1x10^6^ cells and 2 µg of plasmid DNA were used. After transfection, the cells were incubated for 2-3 hours at 37°C in 6-well plates and after a medium change, they were stimulated with 1 µg/ml of lipopolysaccharide (LPS) or 15 U/ml of BLM and mouse lung lysate for an additional 24 h.

### FHL2 knockdown using siRNA

To suppress endogenous FHL2 in fibroblasts, the cells were transfected for 24 h with 5 nM of specific siRNA using the HiPerFect transfection kit from Qiagen (Hilden, Germany). As control, a scrambled RNA probe was used.

### Collection of bronchoalveolar lavage fluid (BALF), sircol collagen and hydroxyproline assays

BALF was collected from euthanized mice after intratracheal application of 0.5-ml PBS supplemented with 2 mM of EDTA. To remove the cells, the samples were centrifuged at 400x*g* for 10 minutes at 4°C. The supernatant was aliquoted and frozen at -80°C until analysis. Approximately 90% of the total instilled volume was consistently recovered. The collagen content in BALF was measured using the Sircol Soluble Collagen Assay Kit (Biocolor Ltd., UK), following the manufacturer's instructions. The hydroxyproline content of left lungs was measured by a colorimetric method according to Edwards and O’Brien [27]. For analysis of immune cells present in BALF, the lung wash procedure was repeated three times and the aliquots were combined.

### Analysis of lung immune status

A single lung cell suspension was obtained by enzymatic digestion. The lung tissue was cut into tiny pieces and incubated in RPMI 1640 medium containing 0.7 mg/ml collagenase A and 100 Kunitz-units/ml DNase I for 60 min at 37°C. The digested tissue was then passed through a “filter-cartridge” to remove large pieces of tissue and centrifuged at 400x*g* for 7-10 min at 4°C. The pellet was resuspended with erythrocyte lysis buffer and immediately centrifuged and resuspended again in FACS-PBS (5% FCS in PBS) for flow cytometry. At least 2x10^6^ cells for each staining analysis were used. Unstained cells were always used as controls. For staining, cells were resuspended and incubated in FACS-PBS containing primary Abs for 20 min on ice, washed twice with FACS-PBS, and further incubated, if needed, with fluorochrome-conjugated secondary antibodies for an additional 20 min. After washing the cells, measurements were performed with a FACS Calibur flow cytometer. The antibodies used were: FITC-rat-anti-mouse CD45.2; FITC-rat-anti-mouse CD11b; biotin-labelled rat-anti-mouse Gr-1 and APC-streptavidin (purchased from BD Biosciences); as well as APC-hamster-anti-mouse CD11c; APC-rat-anti-mouse CD8a, PE-rat-anti-mouse CD4, PE-rat-anti-mouse-MHCII and FITC- or PE-rat-anti-mouse F4/80 and FITC- or PE-rat-anti-mouse Ly6G (obtained from eBiosciences). For intracellular staining with rabbit-anti-S100A9 serum, the fixation/permeabilization kit from BD Biosciences was used. 

### qRT-PCR

From cultured eukaryotic cells total RNA was isolated using the Qiagen RNeasy® Mini kit (Qiagen, Hilden), while from homogenized lung tissue, total RNA was extracted with the TRIzol reagent (Roche Diagnostics, France), in accordance with the manufacturer’s recommendations. RNA integrity was controlled by an Agilent Bioanalyser 2100 (Agilent Technologies, Böblingen, Germany) and transcribed into cDNA using the high-capacity cDNA reverse transcription kit from Applied Biosystems (Darmstadt, Germany). The mRNA levels were determined by TaqMan qRT-PCR using the LightCycler 480 II (Roche Diagnostics, Mannheim, Germany). Each cDNA probe was analysed in triplicate and specific signals were scored in relation to the signals of two housekeeping gene transcripts, Cytochrom C and GAPDH. The primers used were assigned using the Universal ProbeLibrary Assay Design Centre at www.roche-applied-science.com/.

### SDS-PAGE Western blot

RIPA lysates of mouse lung tissues (20 µg/sample) were resolved by 7% or 10% SDS-PAGE and, after electroblotting onto a nitrocellulose membrane, proteins were detected by Western blotting with appropriate antibodies using the ECL detection system. The rabbit-anti-collagen type I antibody was purchased from Merck Chemicals GmBH, Schwalbach, Germany. The rabbit-anti-collagen type III was from Santa Cruz Biotechnology, Inc. and the rat-anti-tenascin C from Abcam Inc., Cambridge, UK. Rabbit-anti-fibronectin, mouse-anti-αSMA and mouse-anti-β actin were from Sigma-Aldrich, Taufenkirchen, Germany. The mAb anti-FHL2, clone F4B2-B11 and rabbit anti-laminin gamma 1 have been described elsewhere [16]. The n-fold of protein expression was performed densitometrically as the relative intensity of the appropriate protein bands to the loading controls. Values at time point 0 were taken as unity. 

### Reporter gene assay

HEK 293 cells were co-transfected with the reporter plasmid pGL3-hTNC-luc (300 ng) and indicated plasmids (1000 ng) using polyethylenimine. Each transfection was carried out in triplicate. Relative light units were measured 24 h after transfection using the Luciferase Assay System (Promega, Madison, WI, USA) and were normalised to protein concentrations of cell lysates. The human TNC promoter comprised a 2000-bp long sequence, including the TATA box and the first 83 bp of the first exon. pCS2+MT-mycFHL2[1/2-4] full length as well as the pCS2+MT-mycFHL2[1/2-2] and pCS2+MT-mycFHL2[3,4] C-terminal and N-terminal truncations containing the LIM domains ½ to 2 and LIM domains 3 to 4, respectively, are described in [28]. Transfection of cells with pcDNA3-HA-MKL1 full-length (FL) or MKL1 truncated dominant-negative (DN) constructs [29] was used as positive and negative control, respectively, for TNC promoter activity.

### Histological analysis

Paraffin sections of lung specimens were deparaffinised and stained according to standard protocols with haematoxylin and eosin or with AZAN trichrome stains according to Heidenhein. The extent of lung fibrosis was determined by morphometry according to the Ashcroft criteria, as described in [30]. Usually, 4 different sections per mouse sample were quantified. All analyses were performed by three independent observers (A.A., C.N. and T.S.) in a blinded fashion. For the immunohistochemistry, paraffin sections were dewaxed, blocked with 10% FBS and incubated with rat-anti-tenascin C (ab6346, Abcam Inc., Cambridge, UK), rat-anti-F4/80 (CI:A3-1, Serotec, Oxford, UK) or rabbit-anti-FHL2 (ab66399, Abcam Inc., Cambridge, UK) antibodies for 1 h at room temperature. A Vectastain ABC-AP Kit from Vector Laboratories was used to visualise the stained proteins. 

### Statistical analysis

Data are expressed as mean+/-SEM unless specified otherwise. Statistical analysis was performed using the Mann-Whitney U test and GraphPad Prism software (version 5) with P values <0.05 regarded as statistically significant and * P<0.05, ** P<0.01, *** P<0.001. In the following N describes independent biological experiments, whereas n accounts for the number of animals analysed per group. 

## Results

### Development of BLM-induced lung fibrosis

**Figure 1:**
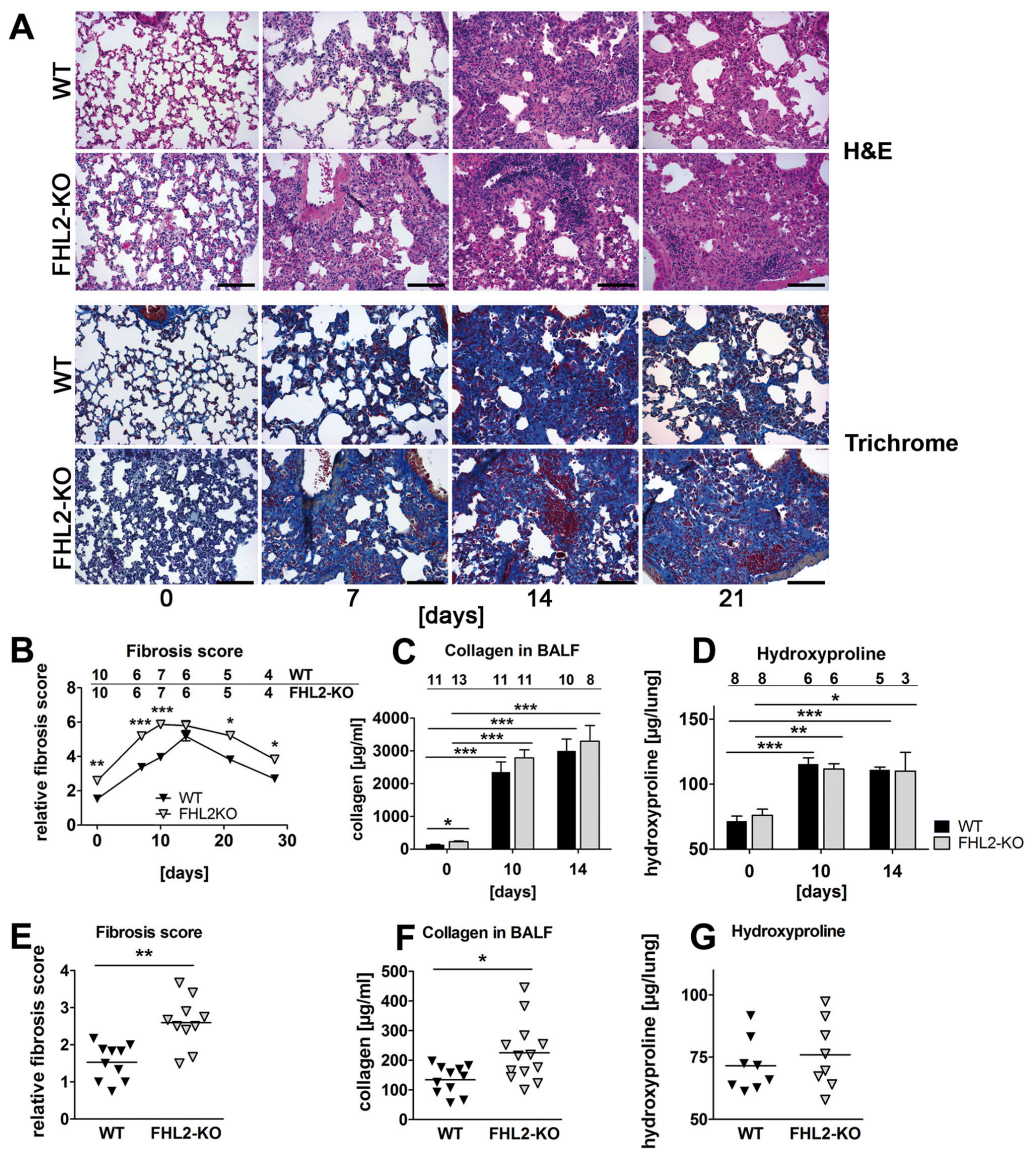
Development of BLM-induced lung fibrosis in FHL2 WT and knockout mice. (**A**) FHL2 WT and knockout mice received a single intranasal application of BLM (2 U/kg) and the right lungs were fixed in 4% paraformaldehyde and embedded into paraffin. Four-micrometre-thick sections were stained with hematoxylin and eosin (upper panel) or with AZAN trichrome according to Heidenhain (lower panel). Bar=200 µm. Representative images from N = 2 and n = 5 to 10 (see B) are shown. (**B**) Lung sections shown in A were scored for fibrotic alterations according to Ashcroft criteria [30] by three observers in a blinded fashion. N = 2. Ciphers above the curves represent the numbers of analysed animals per time point, with upper and lower lines showing WT and FHL2-KO group of mice, respectively. (**C** and **D**) Total collagen amount in the bronchoalveolar spaces (C) and hydroxyproline content in lungs (D) of BLM-treated mice. N = 2 – 3, depending on the time point. Ciphers above the curves represent the numbers of analysed animals per group of mice. (**E** - **G**) Lung fibrosis score (E), collagen amount in BALF (F) and hydroxyproline content (G) in lungs of non-induced control mice.

To analyse the potential role of FHL2 in the development of lung fibrosis, WT and FHL2-deficient mice were first treated with different doses of BLM to compare their sensitivity to the drug. Unexpectedly, the FHL2-KO mice showed a higher mortality rate than WT mice following BLM treatment. At 5 U/kg BLM, almost 50% of FHL2-negative animals died during the first 10 days of drug application, while the vast majority of WT mice survived the treatment (Figure S1A). The higher sensitivity of FHL2^-/-^ mice to the antibiotic was also reflected by the stronger loss and slower recovery of body weight after a single application of BLM at a low dose (Figure S1B). To determine whether the observed differences corresponded with dissimilar pulmonary aberrations, we performed a comparative histological analysis of WT and FHL2-deficient lungs at various time points after BLM application. The H&E staining of lung sections showed a clear increase in the swelling of alveoli and interstitial tissues, as well as an accumulation of infiltrating cells in both WT and knockout mouse lungs (Figure 1A, upper panel). These alterations were transient and reached the maximum in both types of mice two weeks after BLM application, which is in agreement with published data [14]. Staining of serial lung sections with AZAN trichrome showed that the observed alterations were accompanied in both WT and FHL2-null lungs by an increased expression of connective tissue, indicating that the lung changes were mainly of a fibrotic nature (Figure 1A, lower panel). A detailed comparison of lung sections showed that areas occupied by connective tissue were larger in FHL2-KO lungs than in WT lungs, especially at early and late times of BLM administration, suggesting a different progression/regression of fibrotic alterations in the two types of mice. Quantification of lung fibrosis according to the Ashcroft criteria [30] confirmed the difference in the development of fibrosis between WT and KO animals, further revealing that the fibrotic changes were highly significantly different between the two groups at an early time of BLM application, but reached similar levels after two weeks and declined significantly faster in WT mice (Figure 1B). Newly synthesized and deposited collagens represent a large part of connective tissue during fibrosis and quantification of their content is generally used for evaluating tissue fibrosis. The most common methods used thereby are the Sircol Collagen Assay and the hydroxyproline quantification. During lung fibrosis the protein amount per se, and that of collagens in particularly, increases not only in the lung tissue but also in the bronchoalveolar space and changes in their amounts are often used to characterize fibrotic alterations [31–33]. Measuring collagen amounts in bronchoalveolar lavage fluids (BALF) showed a significant increase after BLM treatment in WT and in FHL2^-/-^ mice as well, but the revealed differences between the mouse lines were minimal (Figure 1C). Also at day 10 of BLM treatment no significant differences could be measured, despite large differences in lung morphology, estimated by Ashcroft criteria (Figure 1B). Similar results were obtained when the hydroxyproline content in lungs was used as a measure for fibrotic changes. Also here the hydroxyproline content in 10- or 14-days treated lungs significantly differed from untreated controls but the alterations in both mouse strains were similar. Although both Sircol and hydroxyproline methods revealed similar results, measuring the collagen content in BALF revealed a larger difference between control and BLM-induced mice than the hydroxyproline assay did, suggesting a different sensitivity of these methods. Furthermore, different methods used here for assessment of fibrotic changes showed that evaluation of lung pathology according to Ashcroft criteria after H&E or trichrome staining encompasses more than just alterations in expression or deposition of collagens. Interestingly, our analyses revealed that the lungs of the non-treated control FHL2^-/-^ mice already displayed some fibrotic properties (Figure 1A and 1B). Although a large variability between individual animals was observed (Figure 1E-G), FHL2-KO lungs generally displayed a denser lung structure with a stronger expression of connective tissue in the interstitial spaces (Figure 1A). Ashcroft values (Figure 1E) and the amounts of Sircol-measured collagens in BALF (Figure 1 F) confirmed this observation but not the hydroxyproline measurement (Figure 1G), which might be due to differences in the sensitivity of methods used. 

### Expression of ECM proteins during BLM-induced lung fibrosis

**Figure 2:**
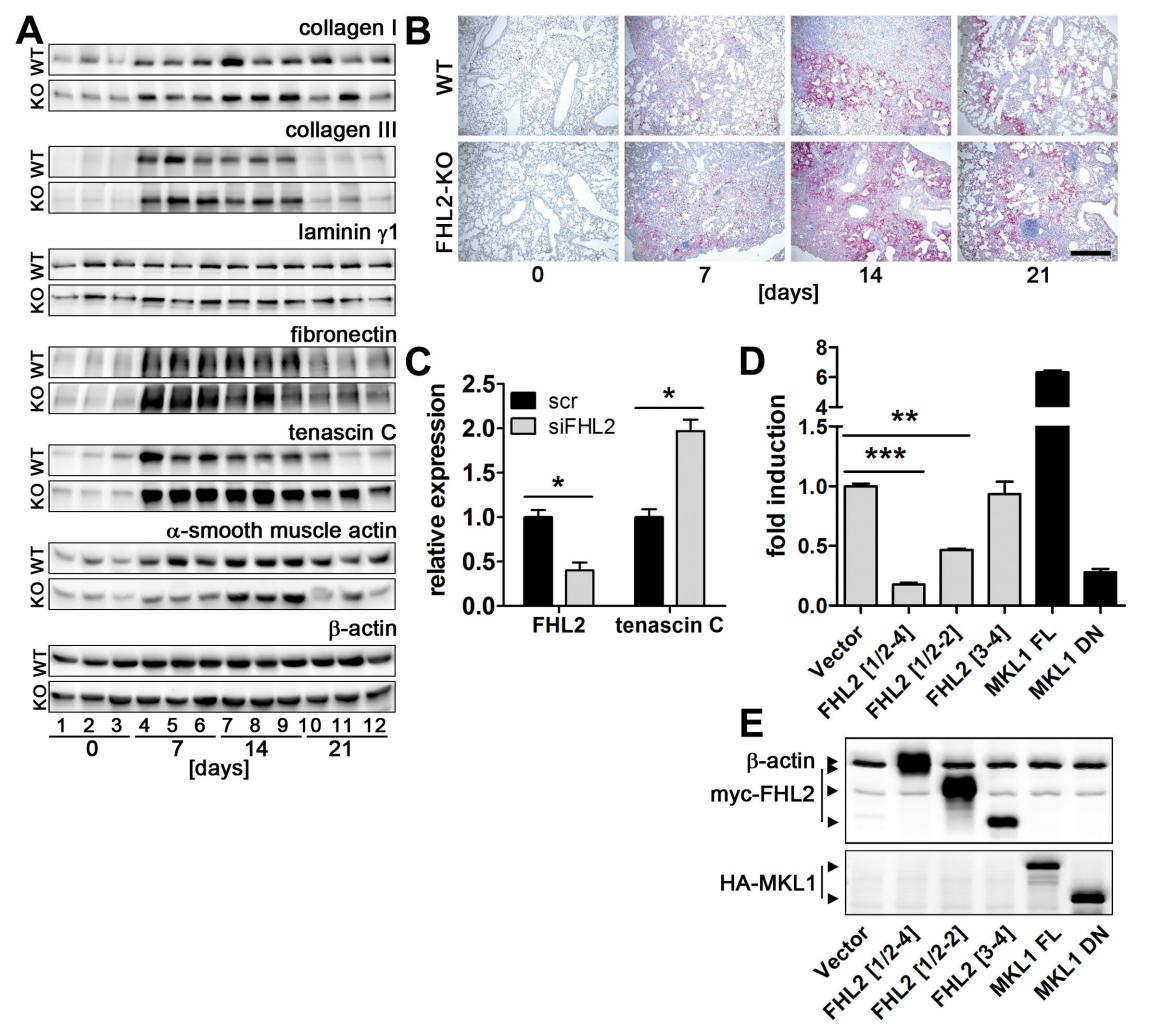
FHL2 inhibits expression of tenascin C. (**A**) FHL2 WT and knockout mice were administered BLM for the indicated times. The left lung was then lysed with RIPA buffer and 20 µg of total lysates were analysed by Western blotting for the expression of different proteins. N = 2, n = 5 to 6 per time point, see Figure S3 for details. Lung lysates from three animals per time point are shown. Equal protein loads were verified by β-actin immunoblotting. (**B**) Paraffin lung sections of control and BLM-induced mice were stained for tenascin C (red). Nuclei (blue) were counterstained with haematoxylin. Bar=500 µm. (**C**) Immortalised embryonal fibroblasts from C57Bl/6 mice were transfected with scrambled or FHL2-specific siRNA for 24 h and the expression of tenascin C examined by TaqMan qRT-PCR. N = 3. (**D**) HEK 293 cells were cotransfected for 24 h with a luciferase reporter gene construct containing a 2000-bp tenascin C promoter sequence and indicated plasmids and the luciferase activity was then measured. Relative scores are presented. Mean values of N = 4 shown. (**E**) The expression of transfected plasmids was verified by Western blotting. A mixture of anti-myc and anti-actin antibodies was used to visualize FHL2 protein and the loading control β-actin on the same blot (upper panel). FHL2[1/2-4] represents the full length protein containing aa 1-279. FHL2[1/2-2] and FHL2 [3,4] represent C-terminal and N-terminal truncations containing the LIM domains ½ to 2 and LIM domains 3 to 4 or aa 1-157 and aa 159-279, respectively, and are described in [28]. MKL1 FL represent the full-length MKL1 protein and MKL1 DN, the dominant-negative MKL1 protein truncated at its N-terminal PPEL motifs and C-terminal transactivation domain, described in [29]. The MKL1 proteins were used as positive and negative control, respectively, for TNC promoter activity.

Fibrotic alterations are usually paired with excessive expression and deposition of different extracellular matrix proteins, not only collagens. Therefore, we compared alterations in activity of genes coding for ECM proteins and alterations in ECM protein amounts in the lungs of WT and FHL2-KO mice after BLM treatment by qRT-PCR and Western blot. Consistent with fibrotic scores shown in Figure 1, an increase in transcriptional activity of ECM genes and an accumulation of matrix proteins was seen in both WT and FHL2-KO mice (Fig. 2A, Fig. S2 and S3). However, no large differences between the two mice strains in the increase of the most analysed transcripts were detected. Nevertheless, a general trend towards a higher deposition of ECM proteins in FHL2-KO lungs was seen, with collagen type III and particularly tenascin C (TNC) showing a stronger and long-lasting appearance (Fig. 2A and Fig. S3A). To confirm these results we compared the amounts of ECM proteins in lungs of WT and FHL2-KO mice, loading samples of both lung types on the same gel. Ten-days-long BLM-treated mice were analysed. Protein quantification of these blot images confirmed the observed trend, namely a higher protein accumulation in FHL2-KO lungs, with collagen type III and tenascin C being the most prominent (Fig. S3B). The stronger and long-lasting presence of tenascin C in FHL2-KO lungs compared with WT lungs was also confirmed by immunohistochemistry (Fig. 2B). Altogether, these data were in good agreement with the histological, Sircol and hydroxyproline analyses. They confirmed that connective tissue proteins were induced in the lungs of both WT and KO mice, with a trend to a stronger deposition and/or slower resolution in FHL2-KO lungs. Specifically distinguishing was the behaviour of TNC, a protein that is known to be upregulated in response to inflammation and tissue remodelling [34], particularly also in the BLM lung fibrosis model [35]. Its expression kinetic in FHL2-KO lungs pointed towards a stronger inflammation at early stages of BLM treatment and a slower halt of inflammatory and tissue damaging processes at later stages. To investigate whether FHL2 is a regulator of TNC expression, we knocked down FHL2 in mouse fibroblasts that spontaneously express high levels of TNC. This indeed led to an increase in TNC transcripts (Fig. 2C), proving thus, that decreased levels of FHL2 increased the levels of TNC transcripts. Furthermore, FHL2 strongly inhibited TNC promoter activity when it was co-expressed with a reporter gene construct containing the tenascin C promoter sequence upstream of a luciferase gene. Using N-terminal and C-terminal FHL2 deletion constructs revealed that the entire FHL2 protein was required for efficient inhibition (Figure 2D and E). Regulation of the tenascin C promoter by full-length and truncated forms of the transcriptional coactivator MKL1 [29] was used as positive and negative control, respectively.

### FHL2-deficient mice exhibit a more pronounced inflammation status in lungs

**Figure 3:**
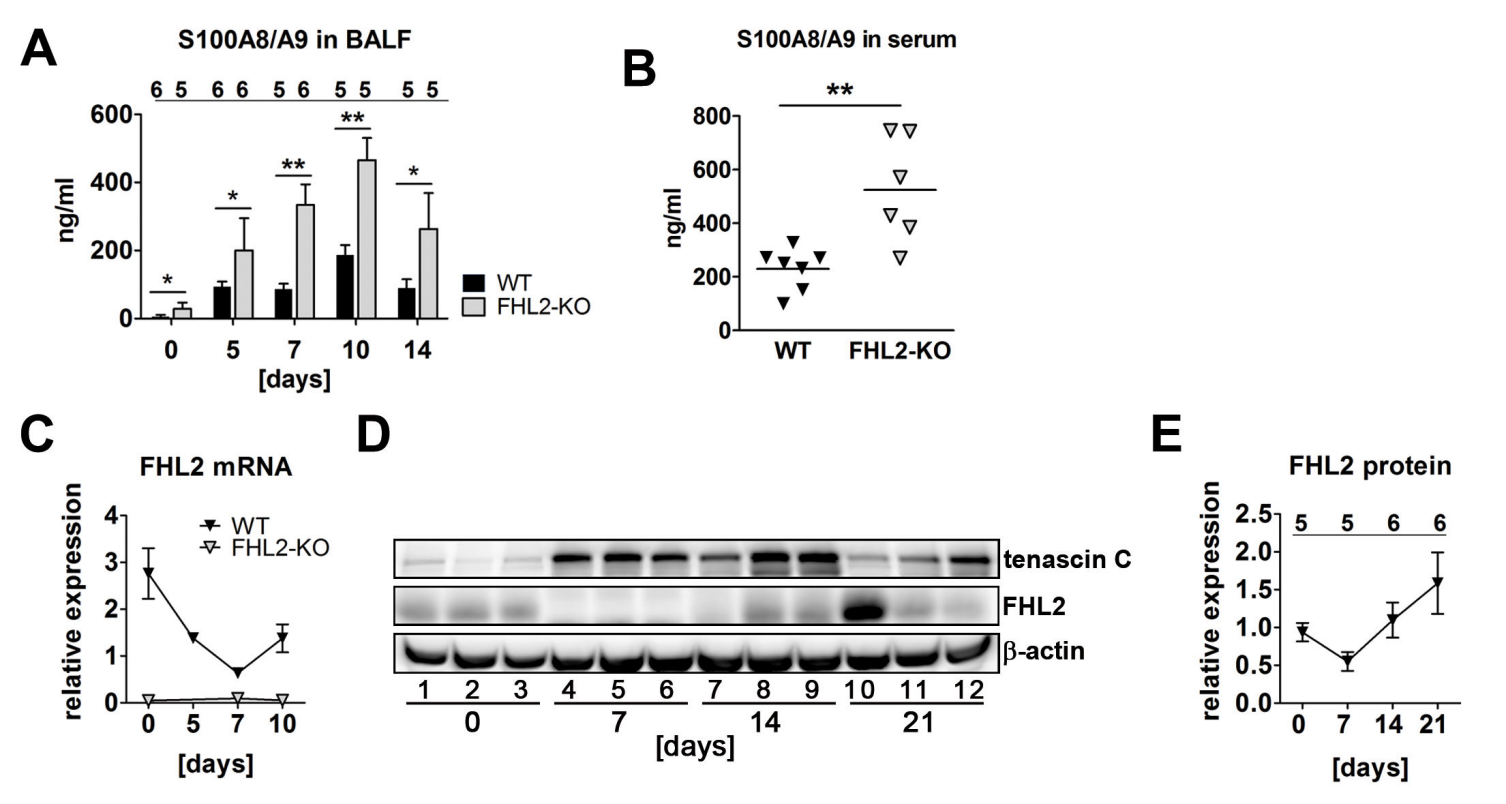
FHL2-deficient mice have a higher inflammation status in the lungs. (**A**) The content of the S100A8/A9 soluble proteins was measured in the BALF of mice by ELISA. The number of analysed animals per time point is shown above the columns. (**B**) Concentration of S100A8/A9 proteins in the serum of control mice measured by ELISA. (**C**) FHL2 transcripts in the lungs of BLM-treated mice were estimated by TaqMan qRT-PCR. The number of animals analysed per time point varied from 6 to 12. (**D**) Western blot analysis of FHL2 expression in the lungs of WT mice after BLM-treatment. Representative images of five to six analysed animals per each time point are shown. (**E**) Quantification of FHL2 WB images. The FHL2 amount was estimated densitometrically as the relative intensity of the FHL2 bands compared to those of the loading controls. Values at time point 0 were taken as unity. N = 2. The number of analysed animals per time point is shown above the curve.

The enhanced expression of tenascin C in FHL2-KO lungs pointed towards a higher inflammation state in the lungs of knockout than in WT animals following BLM induction. To approve this suggestion, we measured the levels of the S100A8/A9 heterodimer protein in bronchoalveolar fluid during the first two weeks of BLM administration, the time amount during which the major pathological alterations in lungs occurred (see Figures 1A and B). The S100A8 and S100A9 are calcium-binding proteins that belong to the group of damage-associated molecular pattern proteins or alarmins. They are released by neutrophils and monocytes very early during inflammation and are increasingly used as biomarkers for ongoing inflammatory processes, as the amount of released S100A8/A9 in circulation or tissues correlates very well with the degree of inflammation [36–39]. Data presented in Figure 3A show that the amount of soluble S100A8/A9 proteins grew rapidly after BLM administration, peaking on day 10 and declining afterwards, suggesting that the major inflammation phase in both types of mice takes place during this time interval. Interestingly, the amount of S100A8/A9 proteins was permanently higher in FHL2^-/-^ mice (Figure 3A). Furthermore, non-stimulated control FHL2^-/-^ mice already displayed significantly higher levels of the S100A8/A9 proteins in the BALF and serum (Figures 3A and B), which is consistent with increased fibrotic scores measured in lungs of intact FHL2-KO mice. Given that the absence of FHL2 facilitates a higher inflammation state and that the BLM-mediated development of lung fibrosis is driven by acute pulmonary inflammation due to the high destruction of lung tissue, it is conceivable that the amount of FHL2 in WT lungs will decrease during lung fibrosis. Indeed, both qRT-PCR and Western blot analyses of FHL2 mRNA and protein showed that its expression was downregulated during the early destructive stage of BLM-treatment, but increased again at later stages (Figures 3C-E). Taken together, our data indicated that the more severe lung phenotype observed in FHL2-deficient mice may be based on the stronger induction of inflammation at the early stages of BLM application. It is remarkable that FHL2 was downregulated in WT mice at this stage and that its expression increased again when the acute inflammation/destruction phase passed on to the tissue regenerative phase. The mode of FHL2 alteration after BLM-treatment was reversal to that of tenascin C (Figure 3D).

### Immune status of bleomycin-treated lungs

**Figure 4:**
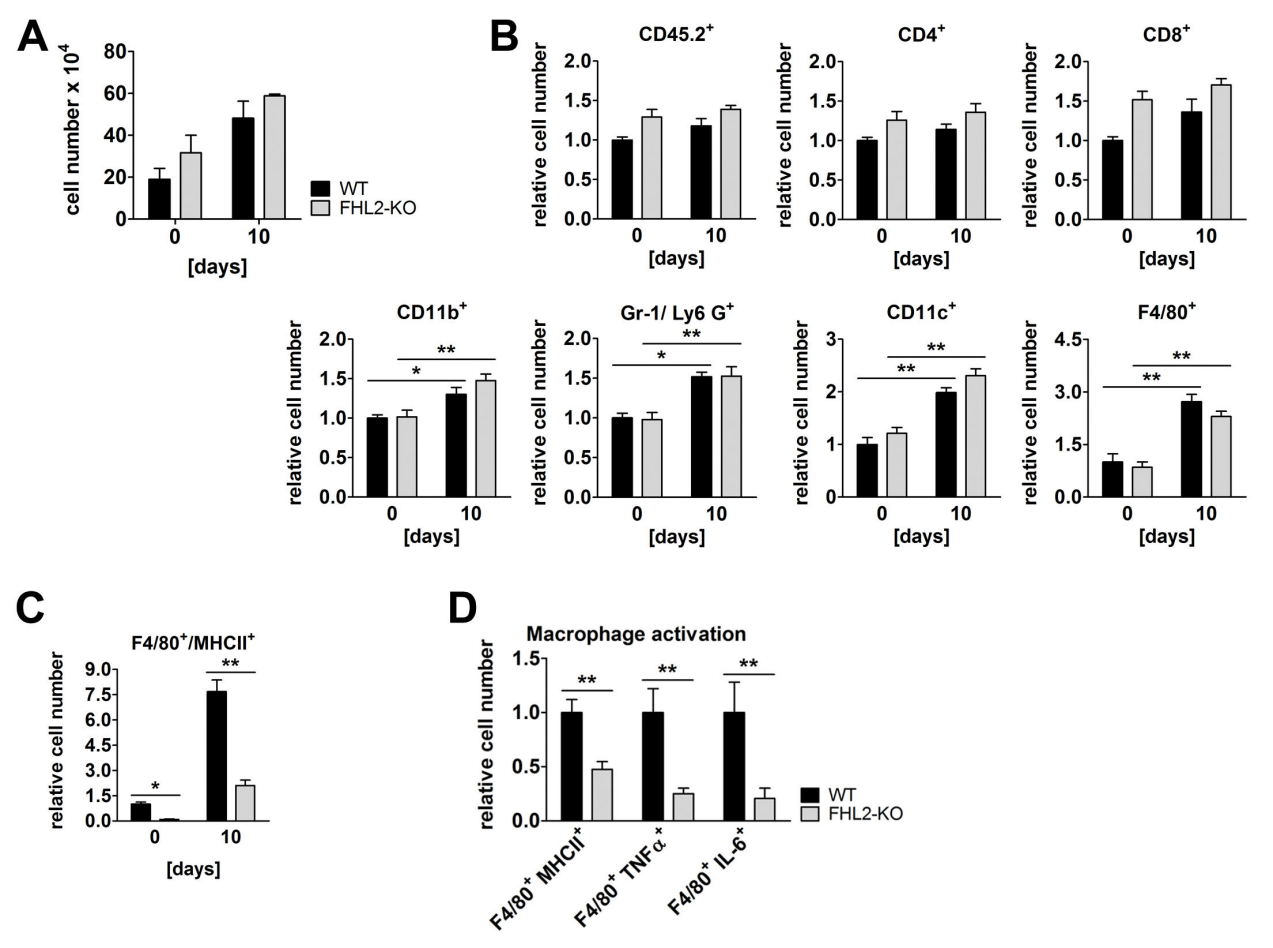
Immune status of WT and FHL2-KO lungs. (**A**) Cell count in BALF of control and 10-day BLM-treated mice. N = 4 and 2 for control and BLM-treated mice, respectively, with 3 to 4 mice per group in each experiment. (**B**) Single cell suspensions of lung tissue from control or 10-day BLM-treated mice were studied for the indicated surface receptors by flow cytometry. A total of 10^5^ cells per lung were analysed. n = 8 and 9 for control and n = 5 and 6 for BLM-treated WT and FHL2-KO mice, respectively. The relative mean amount of immune cells presented in the lungs of wild type mice was always assigned a value of 1. (**C**) Lung cell suspensions from (B) were analysed for MHCII and F4/80 surface marker. (**D**) The F4/80-positive macrophages of BLM-induced mice were further analysed for expression of activation markers: the MHCII receptor and the intracellular TNFα and IL-6 proteins.

To study changes in the immune response to BLM-mediated lung tissue injury, we first analysed the increase of the total amount of immune cells in bronchoalveolar space 10 days after BLM administration, at the peak of inflammation in both mouse strains judged by the content of S100A8/A9 proteins in the BALF (see Figure 3A). The number of extravasated cells increased, as already published [12–14], and was higher in FHL2-KO mice compared to WT mice but not significantly (Figure 4A). Histological analyses showed that the lung-infiltrating cells were predominantly found in fibrotic areas with dense parenchyma and large amounts of deposited extracellular matrix (Figure 1A). Therefore we digested the lung tissue with collagenase and DNase and analysed single lung cell suspensions by flow cytometry. In control mice, no significant differences between WT and FHL2-KO mice in the number of immune cells were seen (Figure 4B). On BLM administration, the number of infiltrated immune cells increased, but only cells of non-lymphoid origin showed a significant increase, with F4/80-positive cells showing the largest accumulation (Figure 4B). While the differences in the amounts of analysed cells between WT and FHL2-KO mice were only marginal, a tendency towards a larger count of all analysed cell types in FHL2-KO lungs was nonetheless present. Macrophages are not only involved in the progression of inflammation during tissue injury, but also in resolution of the inflammation at later phases [40,41]. Thus, we analysed the activation state of infiltrated macrophages. Double staining of lung cells for F4/80/MHCII markers revealed that macrophages of FHL2-KO mice expressed decreased levels of MHCII receptor (Figure 4C). Interestingly, already a lower amount of F4/80-positive macrophages of control FHL2-KO lungs expressed MHCII receptor. A more detailed phenotype analysis of BLM-induced lung macrophages showed that FHL2-deficient cells indeed failed to activate, as they expressed not only low amounts of MHCII receptors but also lower amounts of TNFα and IL-6 (Figure 4D). 

### FHL2 deficiency abrogates DC-SIGN-mediated activation of macrophages

**Figure 5:**
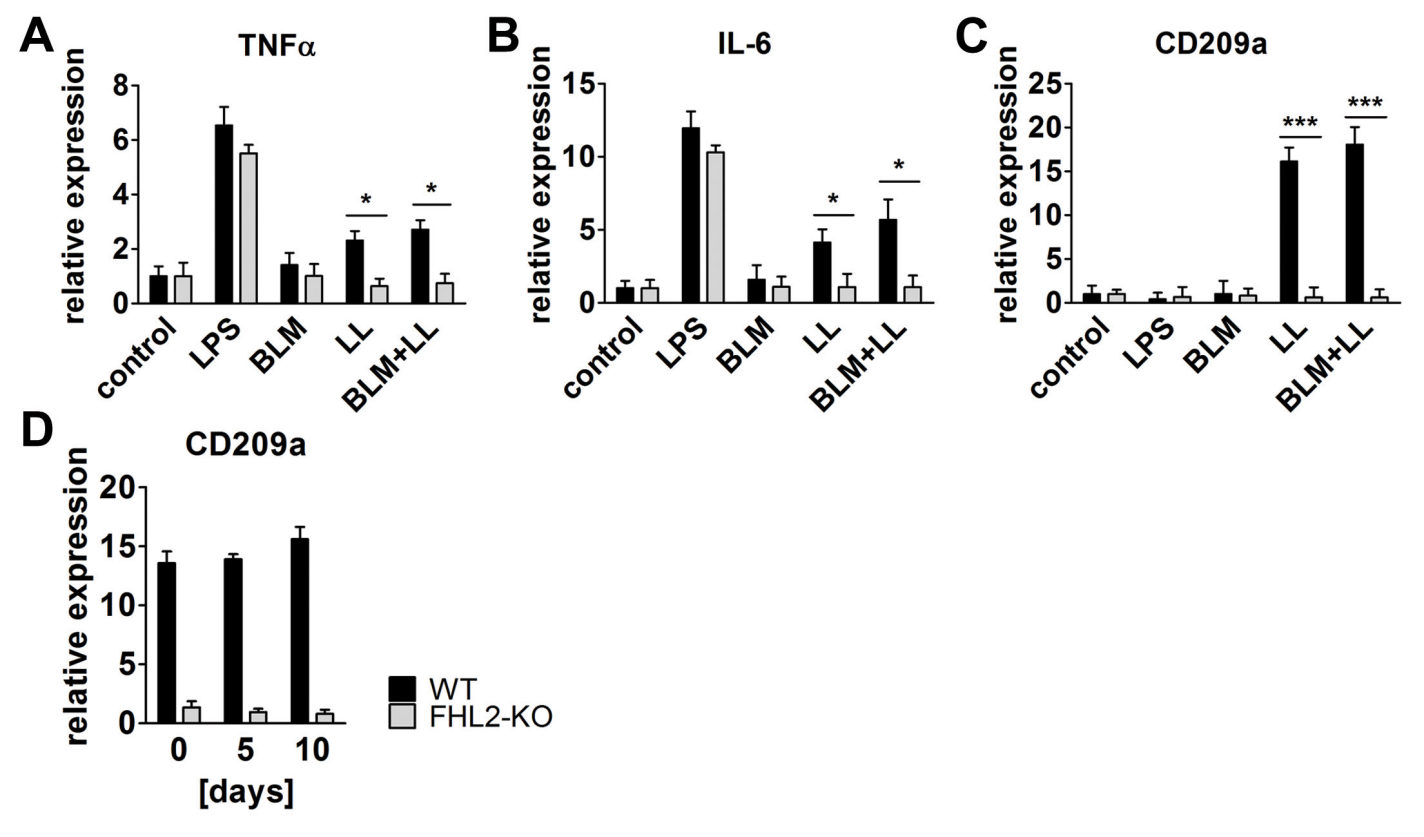
FHL2 deficiency abrogates CD209a/DC-SIGN-mediated activation of macrophages. (**A**-**C**) FHL2 WT or knockout mice were injected i.p. with 0.8 ml of 4% starch in PBS and three days later, peritoneal macrophages isolated from three to four mice were combined and plated into 6-well plate dishes. After incubation for 2-3 h, the non-adherent cells were washed off with PBS and the remaining attached macrophages were stimulated with LPS (1µg/ml), BLM (15 U/ml), lung tissue lysate (LL) or BLM+LL for 24 h. After isolation of total RNA, the transcripts of the indicated genes were determined by TaqMan qRT-PCR. Mean values of three repeated experiments are shown. (**D**) The mRNA levels of D209a/DC-SIGN in the lungs of WT and FHL2-KO mice were determined by qRT-PCR (n=4-5 per group).

**Figure 6:**
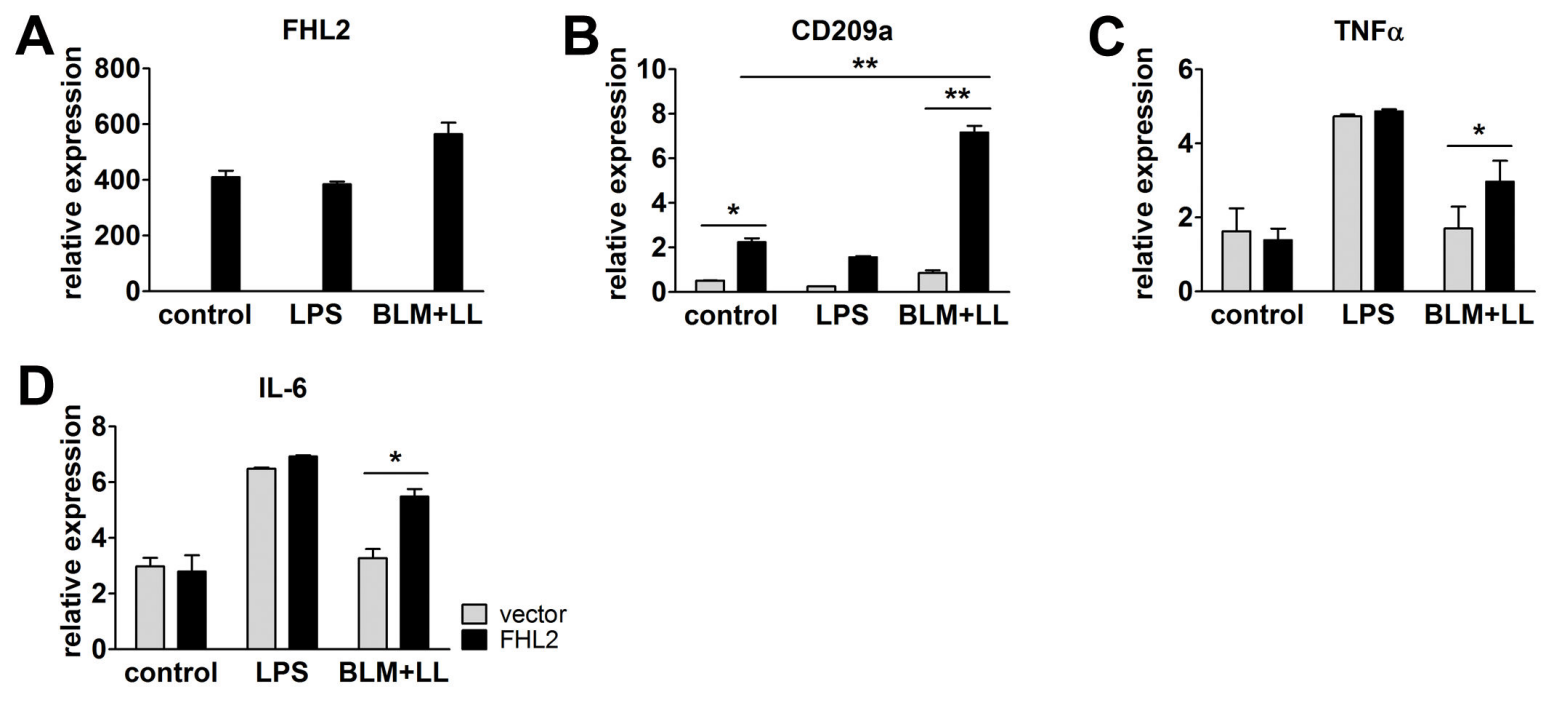
Rescue of FHL2 in FHL2-deficient macrophages restores their ability to upregulate the CD209a receptor and to respond to BLM and lung lysate. Peritoneal macrophages from FHL2-KO mice were transfected with empty vector or human FHL2 cDNA, stimulated with LPS or BLM plus mouse lung lysate, and analysed for expression of (**A**) human FHL2 and (**B**) endogenous mouse CD209a, (**C**) TNFα and (**D**) IL-6 by TaqMan qRT-PCR. N = 3, with macrophage samples being pooled from three to four animals in each experiment.

To study whether the absence of FHL2 inhibited the activation of macrophages in general or the observed effect was specific for BLM-induced lung damage, we isolated peritoneal macrophages from WT and FHL2-KO mice, stimulated them *in vitro* with lipopolysaccharide (LPS), BLM or lung lysate, and analysed them for activation markers. The latter stimuli should imitate the situation in the lung after BLM application (the high lung tissue damage). As the data in Figure 5A and B show, LPS could still activate FHL2-KO macrophages, but lung lysate could not. BLM alone had no effect and it also did not enhance the effect of the lung lysate. LPS is known to stimulate macrophages via binding to the TLR4 receptor [42]. Following lung tissue damage, however, large amounts of carbohydrates are released, which may activate macrophages via the type C lectin CD209a (DC-SIGN) receptor [43]. qRT-PCR analysis of CD209a transcripts revealed only a marginal expression in non-activated WT and KO macrophages and its amount was not changed on LPS stimulation. However, when cells were incubated with lung lysate or lung lysate plus BLM the transcription of CD209a increased dramatically, but only in WT macrophages (Figure 5C). Lung analysis also confirmed the negligible expression of CD209a in FHL2^-/-^ mice, which did not change following BLM treatment (Figure 5D), suggesting that FHL2 is probably involved in the transcriptional regulation of CD209a. To test this, peritoneal macrophages from FHL2-KO mice were transfected with a plasmid containing human FHL2 cDNA and stimulated with LPS or lung lysate plus BLM. Analysis of their phenotype demonstrated that expression of recombinant FHL2, but not the empty vector, rescued the transcriptional deficiency of CD209a following stimulation with carbohydrate-rich lung lysate. As expected, rescue of FHL2 expression had no influence on LPS-mediated activation of FHL2-KO macrophages, but significantly improved macrophage activation by lung lysate and BLM stimulation (Figure 6A-D).

### Macrophage depletion aggravates lung fibrosis

**Figure 7:**
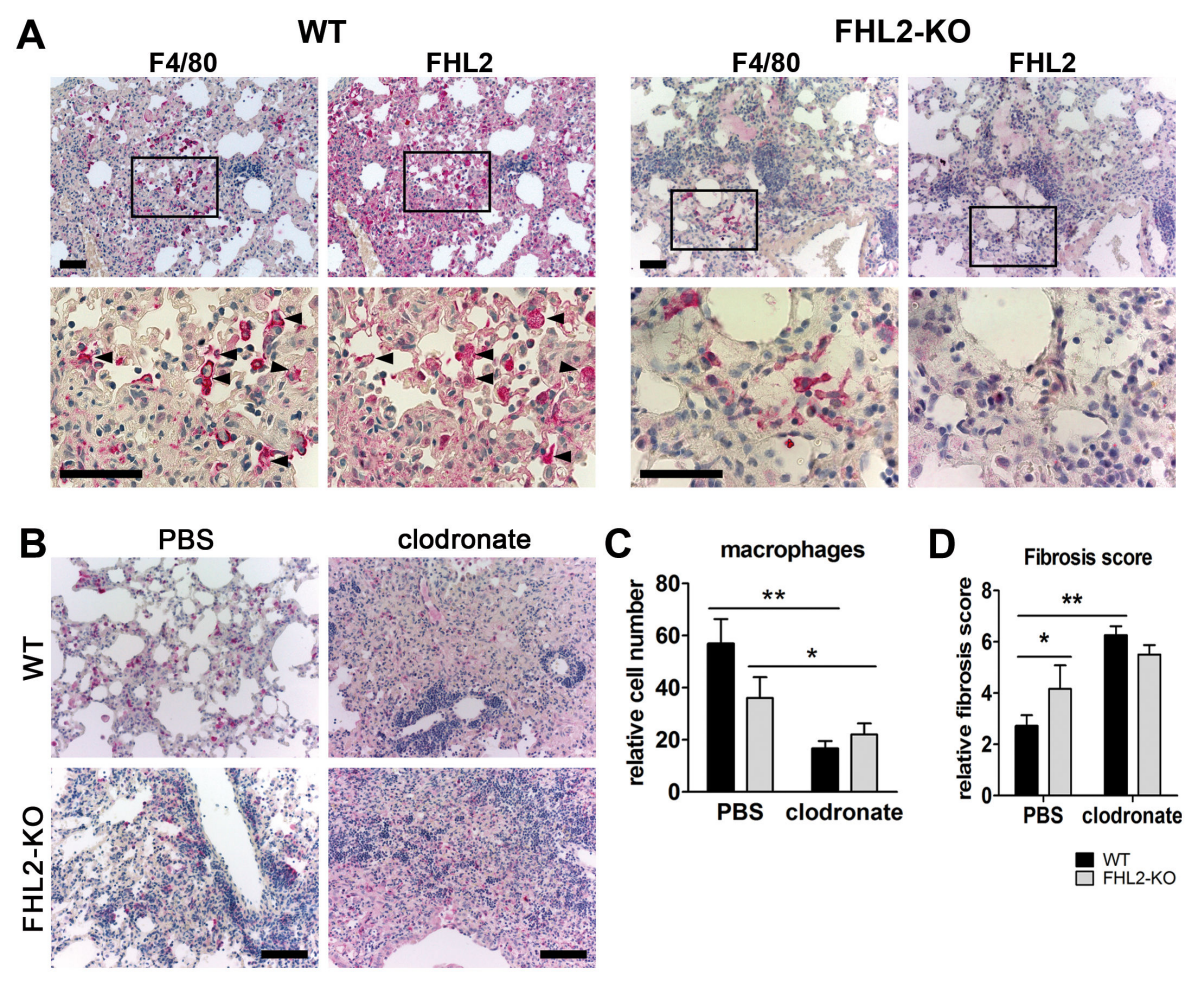
Depletion of macrophages aggravates lung fibrosis. (**A**) Immunostaining of 2 µm-thick serial paraffin lung sections of 10-days BLM-treated WT (left panel) and FHL2-KO (right panel) mice for macrophage marker F4/80 and FHL2. Samples were counterstained with hematoxylin to reveal nuclei (blue). Arrowheads mark several cells with prominent staining of both proteins. Lower images represent larger magnifications of marked areas of upper images. Bars = 50 µm. (**B**-**D**) FHL2 WT and knockout mice were injected i.v. with 100 µl/mouse of PBS- or clodronate-liposomes. The next day, they repeatedly received 75 µl/mouse of PBS- or clodronate-liposomes, but intranasally. On the following day, the mice received 3U/kg of BLM intranasally and 5 days later, they received another dose of PBS- or clodronate-liposomes intranasally. A higher dose of BLM was used to strengthen the acute inflammation and the impact of infiltrated macrophages. On day 10 after BLM application, the right lungs were fixed in 4% paraformaldehyde, embedded into paraffin and analysed for the presence of macrophages by immunohistochemistry using the anti-F4/80 antibody (B) and for fibrotic alterations according to the Ashcroft criteria (D) after H&E staining (Figure S4A). Bars = 100 µm. Representative images of F4/80 stained lung sections are shown in (B) and F4/80-positive cells that were counted in five microscopic fields with a size of 0.75 mm^2^ in each lung sample are presented in (C). Four animals per group were analysed.

To reveal whether macrophages that infiltrate the lung tissue after BLM induction express the FHL2 protein, we stained serial lung paraffin sections of 10-days-long induced WT mice with antibodies to macrophages and FHL2. Images shown in Figure 7A (left panel) indicate that dense lung areas with fibrotic features are infiltrated by macrophages and that macrophages express FHL2. Staining of FHL2-KO lung sections (right panel) confirmed the specificity of the FHL2 expression. Finally, we investigated to what extent the inadequate amount of activated macrophages in the lungs of FHL2-deficient mice accounted for the observed rapid fibrotic changes. Therefore, the macrophage pools of WT and FHL2-KO mice were depleted by injection of clodronate-liposomes (Figure 7B and C) and lung fibrosis was scored after BLM application (Figure 7D and Figure S4). Ashcroft criteria revealed a significant increase in lung fibrosis after macrophage depletion in both WT and FHL2-KO mice, but the degree of fibrosis was similar in the two groups of animals, thus showing that successful activation and recruitment of macrophages into the injured lung was needed to counteract fibrotic changes. 

## Discussion

As an adaptor protein FHL2 is involved in regulation of numerous cellular functions [15] and although it appears to be dispensable for normal development [26,44], deficiency in FHL2 is associated with delayed mesenchymal regeneration. Previously we showed that failure of FHL2 results in delayed wound healing due to impaired collagen contraction and cell migration [24]. Others have shown that FHL2 deficiency impairs bone remodelling [45,46], epithelial-mesenchymal transition, liver regeneration and invasion of tumour cells [17,47,48]. In this study, we showed that the absence of the adaptor protein FHL2 aggravated BLM-mediated lung fibrosis. Not the final extent of fibrotic alteration was increased, but its development at the early acute inflammation phase was augmented and the resolution of the disease at later stages was retarded. This was unexpected, as FHL2 has been reported to positively regulate the expression of collagens type I and III, and αSMA as well as to promote the assembly of ECM proteins [16,22,49]. However, no differences in transcriptional upregulation of ECM genes between WT and FHL2-KO mice, except for TNC, could be measured by us in lungs upon BLM-induced fibrosis, but rather a trend to enhanced deposition of matrix protein and to increased inflammation in lungs of FHL2-KO mice was observed.

While the exact mechanism of BLM-induced lung fibrosis is not fully known, data indicate that an acute lung inflammation plays an important role in this process [3,4]. Following BLM application, a large amount of tissue damage takes place and results in the release of damage-associated molecular pattern molecules that initiate a non-infectious immune response. Neutrophils are the most abundant immune cells that infiltrate into damaged tissue very quickly after injury and are replaced soon by macrophages. Together with neutrophils, macrophages remove the injured tissue debris, release proinflammatory cytokines but also play an important role in halting inflammation and initiating the regenerative processes by releasing cytokine- and chemokine-blunting proteases and other MMPs [40,50]. Here we demonstrated for the first time that FHL2 inhibits the transcription of the ECM protein tenascin C, but encourages the transcription of the C-type lectin receptor DC-SIGN. Both proteins are tightly connected to inflammatory processes. While tenascin C expression is very restricted in normal adult tissues, it is quickly and transiently upregulated during tissue injury or acute phase inflammation and a persistent tenascin C expression is often associated with chronic inflammation [51]. It is known to promote and maintain inflammation and is increasingly used, along with S100A8/A9 alarmins, as a biomarker for ongoing inflammatory processes [42,52]. Given that tenascin C and S100A9 positive signals indicate where inflammation takes place and the level of inflammation intensity, a stronger inflammation in FHL2-KO lungs can be concluded. Tenascin C modulates cell adhesion as well as attraction of immune cells but also stimulates as a TLR4 ligand the production of proinflammatory cytokines like TNF and IL-6 by macrophages [53]. Besides TLRs, C-type lectin receptors contribute essentially to activation of macrophages [43]. They recognize carbohydrates on pathogens or endogenously released carbohydrates during tissue injury and stimulate in concert with TLR4 the NF-κB signalling cascade resulting in production of proinflammatory cytokines [54]. The C-type lectin receptor mediated axis of the NF-κB activation was apparently defective in FHL2-KO macrophages, as they did not express the DC-SIGN receptor. While the TLR4-dependent axis of macrophage stimulation was intact in FHL2-KO cells, judged by LPS stimulation of macrophages, it was obviously not enough for adequate activation of these cells on BLM-treatment, as FHL2-KO lung macrophages expressed reduced amounts of MHCII as well as TNF and IL-6 (Figure 4). Of note, the absence of DC-SIGN and the activation deficiency of FHL2-deficient macrophages could be rescued by overexpression of recombinant FHL2 protein in these cells (Figure 6). The increased amount of tenascin C along with insufficient activation of macrophages in the wounded lung tissue of FHL2-KO mice probably accounts for the increased development and delayed recovery from fibrotic alterations. Macrophages not only produce inflammation-supporting cytokines but also play an essential role during resolution of inflammation. The potential importance of macrophages in development of lung fibrosis and essentially in resolution of the lung pathology was recently demonstrated by Gibbons and colleagues [41]. They showed that depletion of macrophages during the recovery phase of BLM-induced lung fibrosis dampens its resolution. Also in renal fibrosis, depletion of macrophages during the recovery phase of the pathology led to failure of matrix degradation and persistent scarring [55]. Moreover, myocardial wound healing was impaired when macrophages have been depleted [56]. These data are in good agreement with our results showing a delayed recovery of lung pathology in FHL2-KO mice, the macrophages of which exhibit an inadequate activation by carbohydrate related damage-associated molecular pattern molecules. The increase in lung fibrosis following clodronate-liposome-mediated depletion of macrophages further supports this conclusion. We used in our experiments high doses of BLM, which resulted in rapid acute but not chronic inflammation, and although our experiments did not differentiate between the action of macrophages during the acute inflammation and the later resolution phase of lung fibrosis, they are in line with published data and emphasize the importance of macrophages for resolution of fibrotic lung tissue. Furthermore, we provide evidence for the reason of inadequate activation of FHL2-KO macrophages. We showed that FHL2 is essential for transcription of the C-type lectin receptor DC-SIGN on macrophages, the expression of which, in turn, plays a key role in stimulation of macrophages by injured lung tissue (Figures 5 and 6). Interestingly, while this manuscript was under consideration, Dahan and colleagues reported that Kupffer cells of FHL2-KO mice also displayed a reduced activation state, producing less TNF and IL-6 during liver injury resulting in delayed liver regeneration [17].

## Supporting Information

Figure S1 **FHL2-KO mice are more severely affected by BLM treatment than WT mice.** (**A**) WT and FHL2-KO mice received a single intranasal dose of BLM and were scored for mortality rate 10 days later. N = 3, 5 and 2 per BLM doses analysed and cipher over columns show the number of analysed animals per group. (**B**) Body weight alterations of WT and FHL2-KO mice after a single intranasal application (2 U/kg) of BLM. N = 3 and n = 8 to 10 for each group of mice.
(TIF)

Figure S2 **Changes in transcription of genes coding for matrix proteins after BLM treatment.** FHL2 WT and knockout mice were left untreated or administered BLM for 10 days. The left lungs were then lysed and after isolation of total RNA, the transcripts of the indicated genes were determined by TaqMan qRT-PCR. Five to six animals per each group were analysed.
(TIF)

Figure S3 **Changes in matrix protein expression after BLM treatment.** (**A**) FHL2 WT and knockout mice were administered BLM for the indicated times. The left lungs were then lysed with RIPA buffer and 20 µg of total lysates were analysed by Western blotting for the expression of indicated proteins. The β-actin immunoblot served as loading control. Relative protein scores are presented, which were estimated densitometrically as the relative intensity of the appropriate protein bands to the loading controls. N = 2. Ciphers below the β-actin curves represent the numbers of analysed animals per time point, with upper and lower lines showing WT and FHL2-KO group of mice, respectively. Mean levels of untreated mice were assigned a value of 1. (**B**) FHL2 WT and knockout mice were untreated or administered BLM for 10 days and total lysates were analysed by Western blotting for indicated ECM proteins. β-actin served as loading control. The right image represents protein scores of BLM-treated samples shown on Western blots on the left. Relative densitometric intensities of the appropriate protein bands to the loading controls are shown. Mean levels of WT mice were always taken as unity.
(TIF)

Figure S4 **Depletion of macrophages aggravates lung fibrosis.** FHL2 WT and knockout mice were injected i.v. with 100 µl/mouse of PBS-liposomes or clodronate-liposomes. The next day, they repeatedly received 75 µl/mouse of PBS- or clodronate-liposomes, but intranasally. On the following day, the mice received 3 U/kg of BLM intranasally and 5 days later, they received another dose of PBS- or clodronate-liposomes intrasanally. On day 10 after BLM application, the lungs were fixed, embedded into paraffin and stained with haematoxylin and eosin (**A**). Bar = 250 µm. The collagen amount in the BALF of these mice was measured by the Sircol Collagen Assay (**B**). (**C**) The left lungs were lysed and after isolation of total RNA, the transcripts of the indicated genes were determined by TaqMan qRT-PCR. Four animals for each case were analysed.
(TIF)
