# Supplementary figures and images for: The LIM-Only Protein FHL2 Attenuates Lung Inflammation during Bleomycin-Induced Fibrosis
Source: PLoS One. 2013 Nov 18;8(11):e81356. doi: 10.1371/journal.pone.0081356 (PMC3832604; doi:10.1371/journal.pone.0081356)

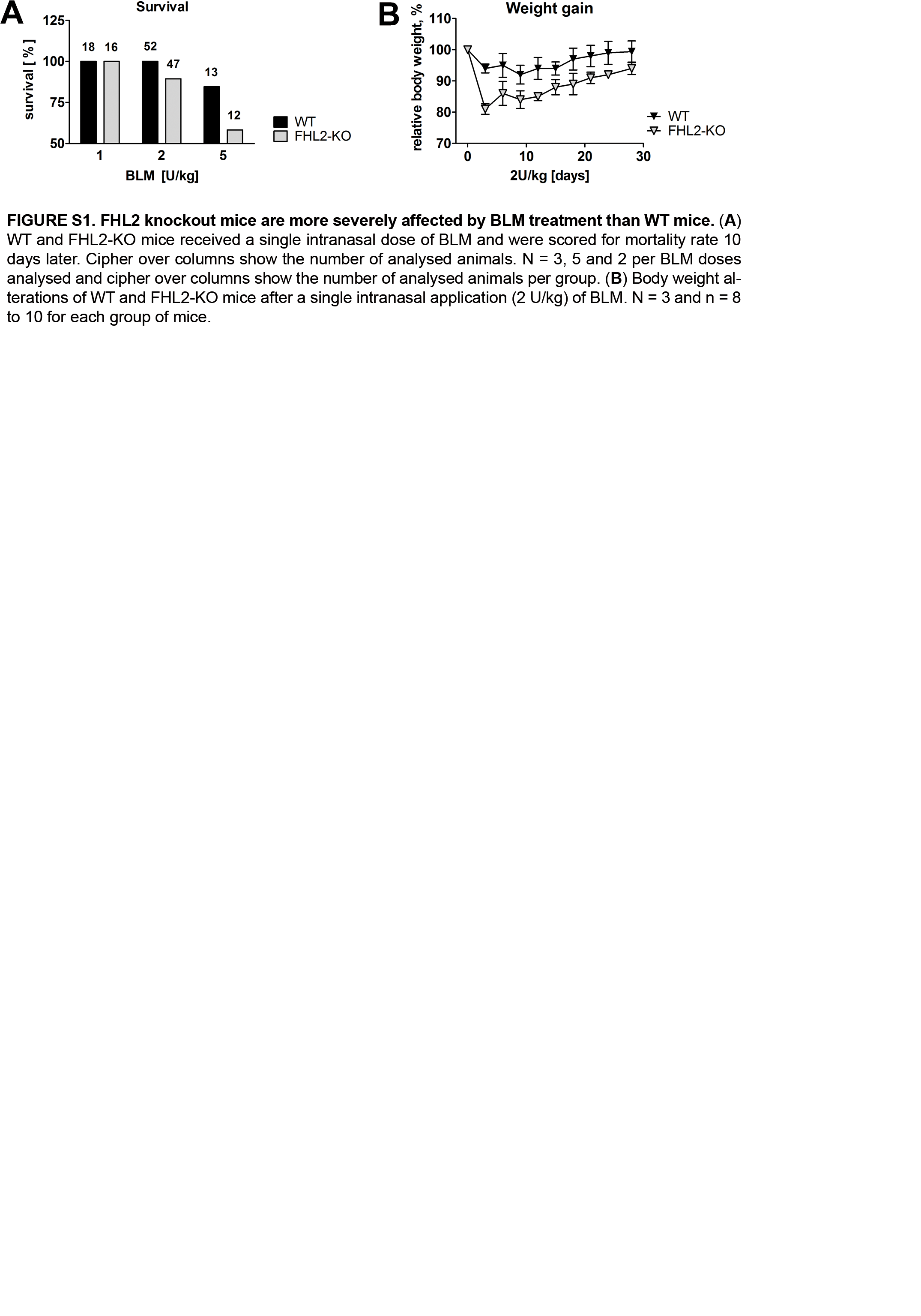

Supplement: Figure S1 — FHL2-KO mice are more severely affected by BLM treatment than WT mice. (A) WT and FHL2-KO mice received a single intranasal dose of BLM and were scored for mortality rate 10 days later. N = 3, 5 and 2 per BLM doses analysed and cipher over columns show the number of analysed animals per group. (B) Body weight alterations of WT and FHL2-KO mice after a single intranasal application (2 U/kg) of BLM. N = 3 and n = 8 to 10 for each group of mice. (TIF) [file pone.0081356.s001.tif]

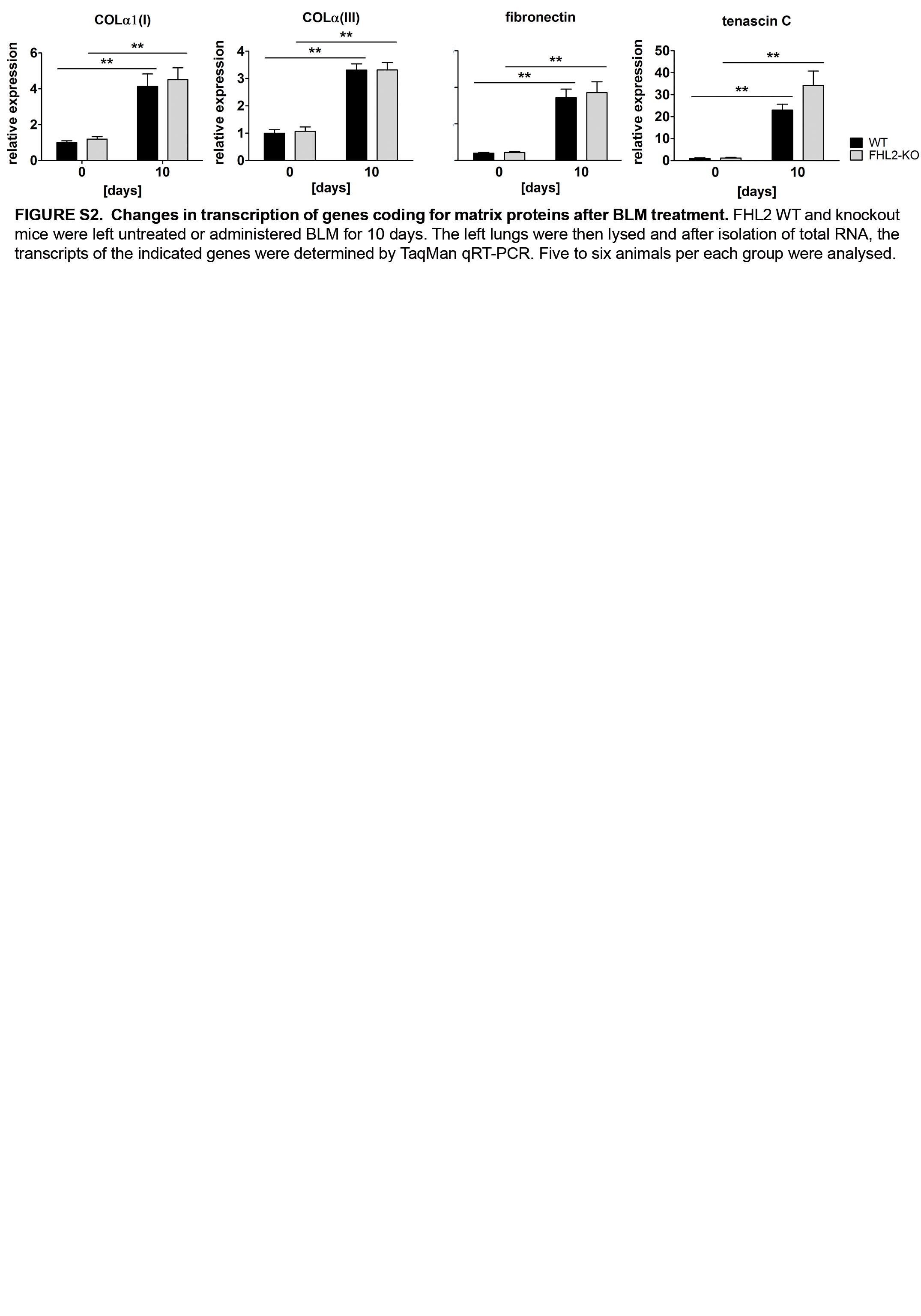

Supplement: Figure S2 — Changes in transcription of genes coding for matrix proteins after BLM treatment. FHL2 WT and knockout mice were left untreated or administered BLM for 10 days. The left lungs were then lysed and after isolation of total RNA, the transcripts of the indicated genes were determined by TaqMan qRT-PCR. Five to six animals per each group were analysed. (TIF) [file pone.0081356.s002.tif]

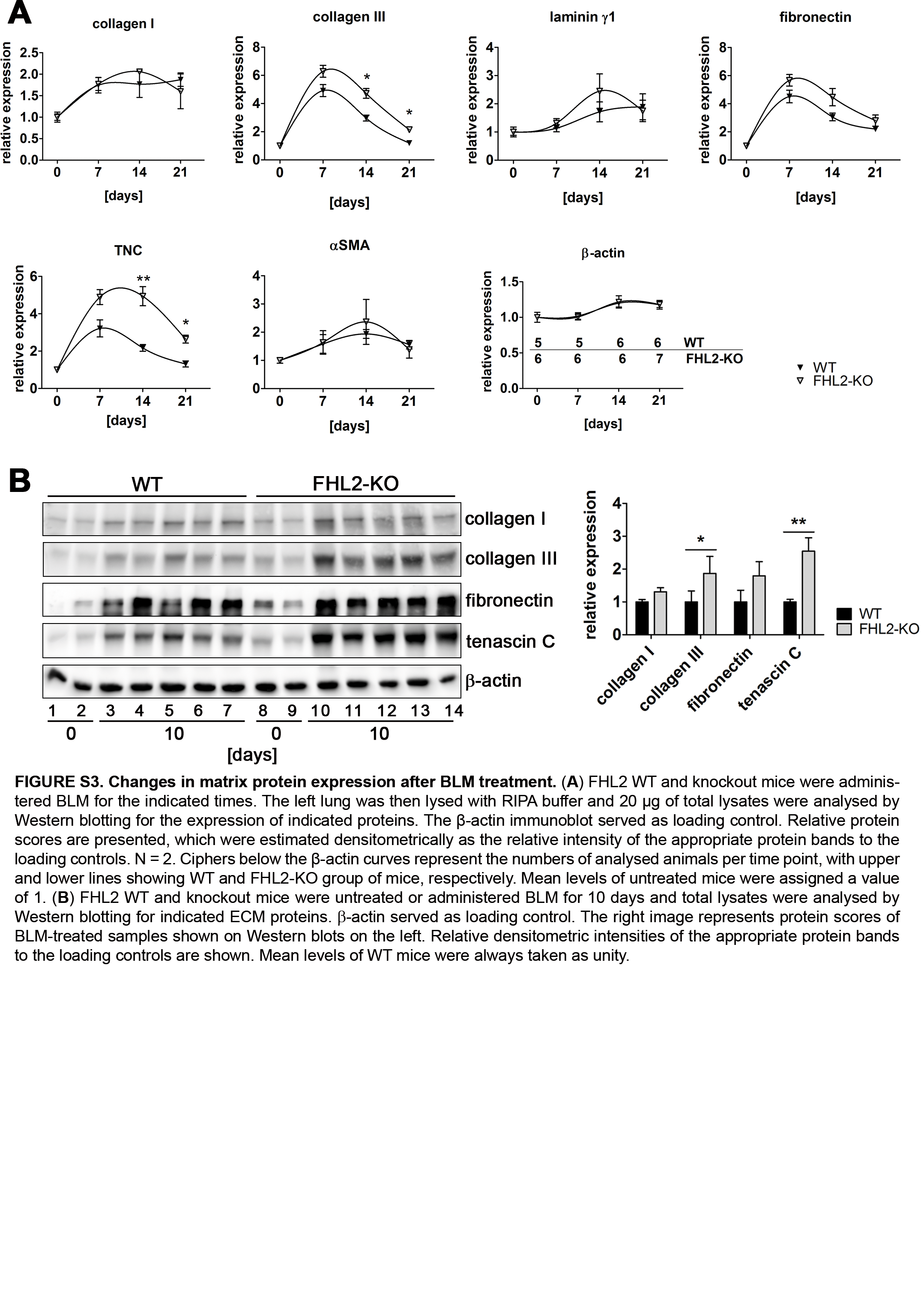

Supplement: Figure S3 — Changes in matrix protein expression after BLM treatment. (A) FHL2 WT and knockout mice were administered BLM for the indicated times. The left lungs were then lysed with RIPA buffer and 20 µg of total lysates were analysed by Western blotting for the expression of indicated proteins. The β-actin immunoblot served as loading control. Relative protein scores are presented, which were estimated densitometrically as the relative intensity of the appropriate protein bands to the loading controls. N = 2. Ciphers below the β-actin curves represent the numbers of analysed animals per time point, with upper and lower lines showing WT and FHL2-KO group of mice, respectively. Mean levels of untreated mice were assigned a value of 1. (B) FHL2 WT and knockout mice were untreated or administered BLM for 10 days and total lysates were analysed by Western blotting for indicated ECM proteins. β-actin served as loading control. The right image represents protein scores of BLM-treated samples shown on Western blots on the left. Relative densitometric intensities of the appropriate protein bands to the loading controls are shown. Mean levels of WT mice were always taken as unity. (TIF) [file pone.0081356.s003.tif]

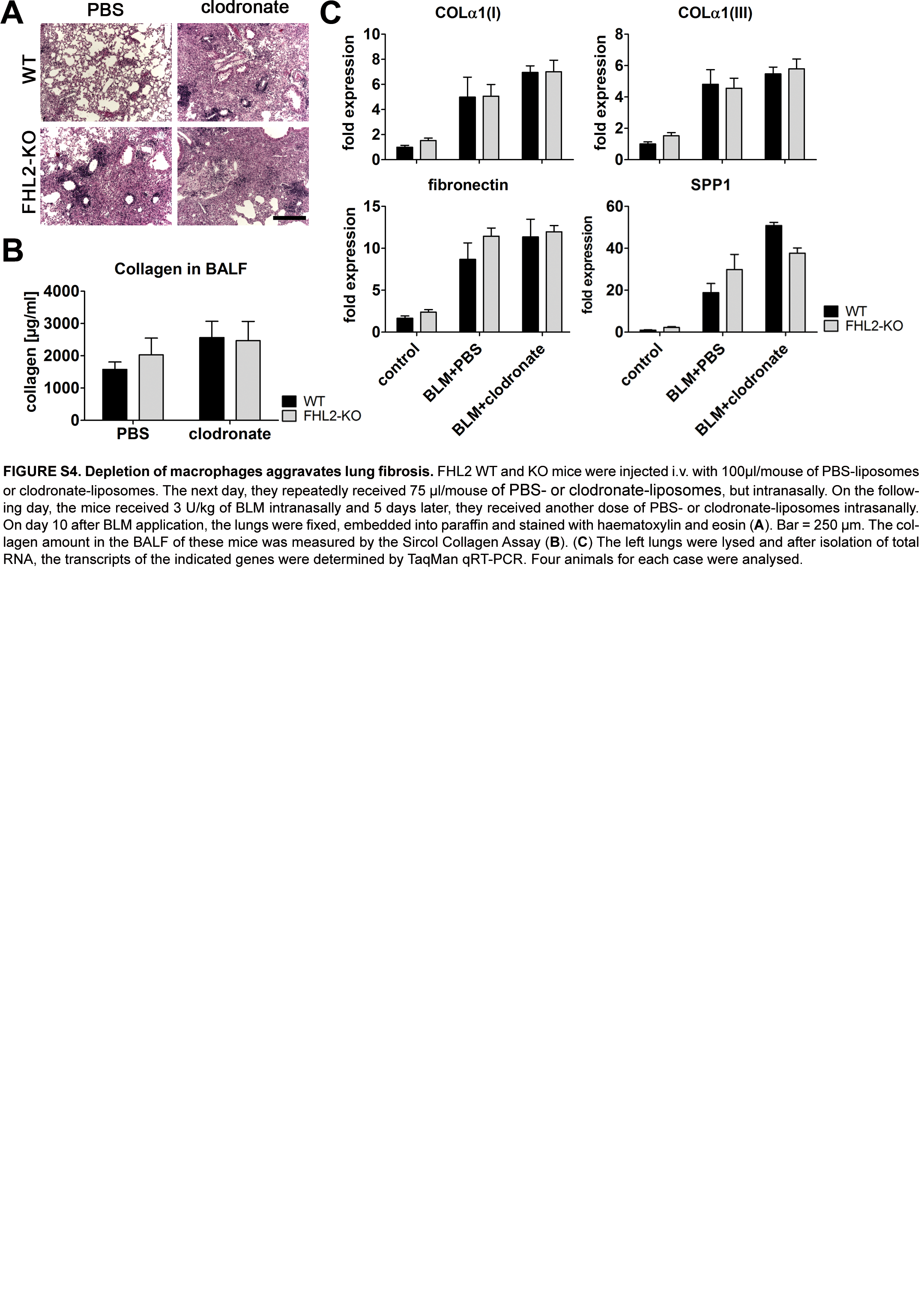

Supplement: Figure S4 — Depletion of macrophages aggravates lung fibrosis. FHL2 WT and knockout mice were injected i.v. with 100 µl/mouse of PBS-liposomes or clodronate-liposomes. The next day, they repeatedly received 75 µl/mouse of PBS- or clodronate-liposomes, but intranasally. On the following day, the mice received 3 U/kg of BLM intranasally and 5 days later, they received another dose of PBS- or clodronate-liposomes intrasanally. On day 10 after BLM application, the lungs were fixed, embedded into paraffin and stained with haematoxylin and eosin (A). Bar = 250 µm. The collagen amount in the BALF of these mice was measured by the Sircol Collagen Assay (B). (C) The left lungs were lysed and after isolation of total RNA, the transcripts of the indicated genes were determined by TaqMan qRT-PCR. Four animals for each case were analysed. (TIF) [file pone.0081356.s004.tif]
